# Role of Ligand Shell Density in the Diffusive Behavior of Nanoparticles in Hydrogels

**DOI:** 10.1021/acs.jpcb.3c03249

**Published:** 2023-10-19

**Authors:** Paige J. Moncure, Jill E. Millstone, Jennifer E. Laaser

**Affiliations:** Department of Chemistry, University of Pittsburgh, Pittsburgh, Pennsylvania 15260, United States

## Abstract

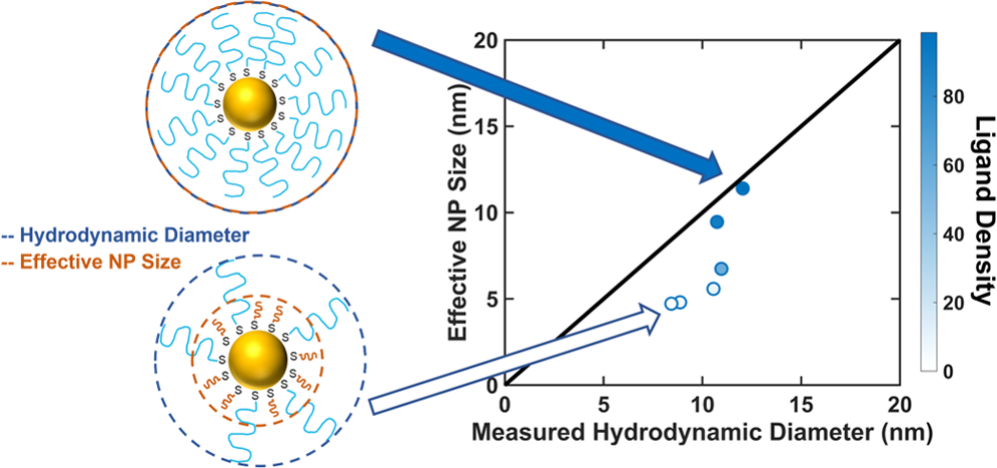

The diffusion coefficients of poly(ethylene glycol) methyl ether thiol (PEGSH)-functionalized gold nanoparticles (NPs) with different effective grafting densities were measured in polyacrylamide hydrogels. The NP core size was held constant, and the NPs were functionalized with mixtures of short oligomeric ligands (254 Da PEGSH) and longer (either 1 or 2 kDa PEGSH) ligands. The ratio of short and long ligands was varied such that the grafting density of the high-molecular-weight (MW) ligand ranged from approximately 1 to 100 high-MW ligands/NP. The diffusion coefficients of the NPs were then measured in gels with varying average mesh sizes. The measured diffusion coefficients decreased with higher MW ligand density. Interestingly, the diffusion coefficients for NPs with high effective grafting densities were well-predicted by their hydrodynamic diameters, but the diffusion coefficients for NPs with low effective grafting densities were higher than expected from their hydrodynamic diameters. These results suggest that crowding in the NP ligand shell influences the mechanism of diffusion, with lower grafting densities allowing ligand chain relaxations that facilitate movement through the gel. This work brings new insights into the factors that dictate how NPs move through hydrogels and will inform the development of models for applications such as drug delivery in complex viscoelastic biological materials.

## Introduction

Nanoparticle (NP) diffusion in a hydrogel matrix is relevant to many fields including polymer physics,^1−4^ materials science,^5−7^ and medicine.^8−18^ For example, NPs have been developed for a myriad of biological applications including imaging,^9−11^ drug delivery,^12−16^ and photothermal cancer therapies.^17,18^ All of these applications rely on NP diffusion in the dynamic chemical and physical environments of living systems, which is difficult to predict due to their inherent complexities.^19,20^ Hydrogels are an attractive model system for studying the interactions that govern diffusion in these complex materials because they have highly tunable chemistries and architectures that can be used to mimic the physical nature of intricate biological environments.^21−23^ Additionally, they are biocompatible and sensitive to external stimuli, making them a promising material for targeted drug release.^24−26^ As such, understanding how the physical features of both hydrogels and NPs influence diffusion in these complex environments is critical for developing the fundamental insight necessary for these applications.

One of the most important features governing the diffusion of NPs in hydrogels is NP size. NP diffusion in hydrogels slows as the diameter of the NP approaches the mesh size of the gel because the NPs become confined by the network. A number of theoretical models have been developed to understand the diffusion of NPs in hydrogels at this scale.^27−35^ Early models considered the network to be composed of immobile polymer chains that NPs had to diffuse around, moving through the pores of the material.^28^ Later models considered interactions between the NP and the polymer, and included polymer chain relaxation as an important factor facilitating diffusion.^29^ Most recently, Cai and Rubinstein proposed a hopping model to explain the diffusion process of NPs that are confined in gels with a mesh size of the same order as the NP diameter. In this model, the NP is trapped in a network cage until thermal fluctuations of the surrounding polymer chains allow for the NP to “hop” through to the next confinement cage.^31^ The NP diffusion is thus dictated by the relaxation time scales of the polymer chains in the network. Together, these theoretical frameworks have laid the groundwork for understanding NP diffusion in hydrogels. However, many complexities of real chemical and biological systems are not yet reflected in these models, and further work is needed on how they should be accounted for.

The theoretical models described above, for example, all treat the NP as a solid sphere. In practice, however, NPs are diverse in their compositions,^36^ sizes,^37^ shapes,^38,39^ and surface chemistries.^40−42^ They are also typically functionalized with small molecules and/or polymers that form a ligand shell around the NP core.^43^ These ligand shells are critical for ensuring that the NPs remain colloidally stable^44,45^ and can impart desirable functionality to the NPs, such as stimuli responsiveness or specific sites for molecular recognition and/or targeting.^46^ However, these ligand shells also increase the chemical and physical complexities of the NPs, especially in applications requiring mixtures of ligands with different sizes and functionalities.^47^ This complexity may, in turn, influence the diffusion mechanisms of NPs in hydrogels. For example, mixing longer and shorter ligands in the same ligand shell may yield particles with a low effective grafting density of the higher-molecular-weight ligands. We recently reported that the diffusion coefficients of NPs with densely packed ligand shells of 1, 2, or 5 kDa poly(ethylene glycol) (PEG)–thiol in polyacrylamide gels appeared to be well-predicted by the hopping model, where the particle size was equal to the hydrodynamic diameter of the NPs.^48^ This result suggested that the two-component NPs were behaving as hard spheres even when the ligand shells made up more than half the particle diameters. This behavior is consistent with simulations of polymer-grafted NPs in polymer melts, where high grafting densities are found to drive the formation of a nondraining layer of hydrodynamically coupled chains around the core of the particle, which increase its effective size.^40,49,50^ However, the same simulations of polymer melts predict that at lower grafting densities, polymer-grafted NPs behave as if they are closer in size to the bare NP as fluctuations of the grafted chain conformations become fast enough to facilitate diffusion through the surrounding melt. While hydrogels are distinctly different from melts because they are covalently cross-linked and swollen with water, we hypothesize that a similar phenomenon may be at work in these systems and that reducing the grafting density of the higher-MW ligands may increase their conformational flexibility and facilitate faster diffusion in crowded hydrogel environments.

Here, we test this hypothesis by preparing gold NPs functionalized with mixtures of shorter (254 Da) and longer (1 or 2 kDa) poly(ethylene glycol) methyl ether thiol (PEGSH) ligands and investigate their diffusion in polyacrylamide hydrogels. Varying the ratio of the short and long ligands allows us to vary the grafting density of the higher-MW ligands (which we refer to as the “effective grafting density”) while maintaining the colloidal stability of the NPs. Diffusion measurements reveal that the diffusion constants of the NPs with high effective grafting densities are well-predicted by their hydrodynamic diameters. The diffusion coefficients of the NPs with low effective grafting densities, on the other hand, are significantly faster than those predicted from their hydrodynamic diameters, suggesting that the lower effective grafting densities enable ligand conformational fluctuations that facilitate transport through the gel. This work provides new fundamental insights into the factors dictating NP diffusion in complex environments and lays the foundation for understanding how surface functionalization, and, in particular, the presence of complex multicomponent ligand shells, affects diffusion processes in confined environments relevant to the development of NP biotherapeutics.

## Experimental Section

### Materials

Gold(III) chloride trihydrate (HAuCl_4_·3H_2_O ≥99.9%), acrylamide (≥99%), ammonium persulfate (APS, 98%), *N*,*N*′-methylenebis(acrylamide) (≥99.5%), *N*,*N*,*N*′,*N*′-tetramethylethylenediamine (TEMED), acetonitrile (ACN, ≥99.9%), sodium borohydride (NaBH_4_ ≥ 99.99% trace metal basis), deuterium oxide (D_2_O ≥ 99.9%), HCl > 99.999% trace metal basis, HNO_3_ > 99.999% trace metal basis, and Aldrich ColorSpec disposable NMR tubes were obtained from Sigma-Aldrich (St. Louis, MO). Poly(ethylene glycol) methyl ether thiol (MW = 254 Da, 1 kDa, 2 kDa) was obtained from Fisher Scientific (Hampton, NH). A Mono Zelux CMOS camera (CS165MU), 12 mm Machine Vision Lens (MVL12M23), and C-mount Adapter (SM1A10) were all purchased from Thorlabs (Newton, NJ). All reagents were used as received unless otherwise indicated. NANOpure (Thermo Scientific, ≥18.2 MΩ) water was used to prepare all aqueous solutions. Prior to use, all glassware and Teflon-coated stir bars were washed with aqua regia (3:1 ratio of concentrated HCl to HNO_3_) and rinsed with copious amounts of water prior to drying. ***Caution**: aqua regia is highly toxic and corrosive and should only be used with proper personal protective equipment and training. Aqua regia should be handled only inside a fume hood.*

### Nanoparticle Synthesis and Characterization

#### 5 nm Gold Nanoparticle Synthesis

Gold NPs were synthesized following a modified Murphy synthesis.^51^ In a 20 mL glass vial, 14.5 mL of water was combined with 500 μL of an aqueous 20 mM gold(III) chloride trihydrate solution. An ice-cold 0.1 M NaBH_4_ solution was prepared. To the gold solution, 0.6 mL of NaBH_4_ solution was added while stirring. Upon addition, the solution color changed from colorless to orange. The mixture was removed from the stir plate and allowed to age for 1 h prior to ligand exchange.

To the as-synthesized 5 nm gold NPs, the combined 1 kDa PEGSH and 254 Da PEGSH ligand solution or the combined 2 kDa PEGSH and 254 Da PEGSH ligand solution was added while stirring. For exact concentrations of the ligand used, see the Supporting Information (SI), Tables S2 and S3. Upon addition, there was a slight color change to a pink/orange color. NPs were aged on the benchtop for 1 h prior to purification.

NPs were separated from excess PEGSH, metal, and salts using molecular-weight cutoff centrifugal filters. Approximately 4.5 mL of each NP solution was transferred to Amicon Ultra-4 Ultracel 30 kDa molecular-weight cutoff centrifugal filters (Merck Millipore Ltd.) and was spun using an Eppendorf 5804R centrifuge with a swing bucket rotor (A-44-4, Eppendorf, Inc.) with a force of 4000 rcf for 10 min. The resulting concentrated NPs (typically 50–100 μL in water) were diluted in the tube to a volume of 4 mL with water. The loose pellet was resuspended by gentle mixing using a pipet prior to recentrifugation. This washing procedure was repeated 4 additional times.

#### Nanoparticle Core Size Characterization

To determine the core size of the NPs, transmission electron microscopy (TEM) was performed on each NP sample. A dilute solution of NPs was made by adding 10 μL of NPs (at as-synthesized concentration, post ligand exchange) to 100 μL water. A 7 μL aliquot was drop-cast onto a carbon type A 200 mesh copper grid (Ted Pella). The sample was allowed to air-dry for at least 5 h and then dried under vacuum overnight. TEM characterization of the particle core sizes was performed on a Hitachi 9500 ETEM with a Gatan Orius camera (Petersen Institute of Nanoscience and Engineering, Pittsburgh, PA).

### Ligand Density Quantification

The grating density of both the higher-MW ligands and the short stabilizing ligands for each sample was calculated from the concentrations of the ligands and NPs in the NP solutions. The ligand and NP concentrations were obtained by ^1^H NMR and inductively coupled plasma optical emission spectroscopy (ICP-OES), respectively, as described below.

#### ^1^H NMR Analysis of Ligand Shell Composition

The composition of the ligand shells was quantified by ^1^H NMR, following a procedure adapted from a previously published method.^52^ NMR samples were first solvent-exchanged by centrifugal purification of NPs three times in D_2_O to remove the residual water. A 50 μL aliquot of the concentrated NP sample was then digested with 1 drop (∼5 μL) of concentrated aqua regia (prepared using HCl > 99.999% trace metal basis and HNO_3_ > 99.999% trace metal basis). All samples were allowed to digest overnight, after which 5 μL of dilute ACN was added to each sample as an internal standard. Samples were finally diluted with D_2_O to a volume of 500 μL before measurement. All NMR measurements were performed on a Bruker 600 Ultrashield magnet with AVANCE III 600 Console (Bruker Biospin, Billerica, MA) at 298 K. For all experiments, a *T*_1_ time of 5 s was used (3× longer than the longest *T*_1_ measured for PEGSH^52^).

The unknown ligand concentrations were determined by comparison to three 5-point standard curves, one for each molecular weight of PEGSH ligand. Standard ligand solutions were prepared in the range of 1.00–0.01 mM ligand (1.00, 0.50, 0.10, 0.05, and 0.01 mM, prepared in D_2_O). To each standard and each sample, 5 μL of dilute ACN (0.24% v/v; 15 μL of ACN in 6.00 mL of D_2_O) was added as an internal reference. The integrals of the PEGSH peaks were normalized to the intensity of the ACN peak. Calibration curves were constructed using the integrals of the backbone protons (3.65 ppm) and the end-group protons (3.33 ppm), as described in detail in the Supporting Information. For each sample, the integrals of the backbone peak and the end-group peak were similarly normalized to the intensity of the ACN peak. The integral of the end-group peak was compared to the end-group calibration curve to determine the total number of PEGSH ligands in the sample. The intensity of the backbone peak was then used to determine the relative numbers of the short and long chains in the sample. Full calibration curves and analysis for mixed ligand shell quantification can be found in the SI.

#### ICP-OES Analysis

Inductively coupled plasma optical emission spectroscopy (ICP-OES) was used to determine the metal concentration for each sample, which was then used to determine the concentration of NPs. For ICP-OES analysis, NP samples were taken from the digested and diluted NMR samples, as described above. From each NMR sample, 200 μL was further diluted to 3 mL using 5% ultrapure aqua regia solution (Sigma-Aldrich, HCl > 99.999% trace metal basis; HNO_3_ > 99.999% trace metal basis) and analyzed via ICP-OES to determine metal concentration. ICP-OES was performed using an argon flow and an Agilent 5100 VDV ICP-OES instrument (Department of Civil and Environmental Engineering, University of Pittsburgh). Unknown metal concentrations were determined by comparison to a 7-point standard curve with a range of 0.10–10 ppm of each metal (0.10, 0.50, 1.0, 2.5, 5.0, 7.5, and 10 ppm) prepared by volume using ICP standards (Fluka, TraceCERT 1000 ± 2 mg/L metal in HNO_3_), all diluted in a 5% aqua regia matrix. All standards and unknown samples were measured 3 times and averaged. A 3 min flush time with a 5% nitric acid matrix was used between all runs, and a blank was analyzed before each unknown sample to confirm the removal of all residual metals from the instrument. The metal concentration was finally converted to the NP concentration using the average NP diameter obtained from the TEM analysis described above.

#### Hydrodynamic Diameter Characterization

The NP diameters were determined by using dynamic light scattering (DLS) measurements. To a clean, dust-free, glass cuvette, 10–100 μL of NP solution was added and diluted to 1 mL with water filtered using a 0.2 μm GHP membrane syringe filter. DLS measurements were taken on an Anton Paar Litesizer operating at a wavelength of 633 nm and a scattering angle of 90°. For each NP sample, multiple NP concentrations were measured to ensure that the measured diameters were not influenced by multiple scattering events.

### Gel Synthesis and Characterization

#### Polyacrylamide Gel Synthesis

Aqueous solutions of acrylamide and bis(acrylamide) were prepared at the concentrations listed in Table 2. To each aqueous solution, ammonium persulfate (10 μL at 10 w/v % per mL of solution) and TEMED (1 μL per mL of solution) were added and mixed thoroughly to initiate cross-linking. Samples were immediately transferred to disposable NMR tubes and capped. Samples gelled within 1 h of initiator addition and were allowed to equilibrate for 12 h before NPs were deposited on top. Gel samples are referred to by the average mesh size determined using small-amplitude oscillatory shear rheology, as described below and summarized in Table 2.

#### Gel Characterization

Rheometry was performed on an Anton Paar MCR 302 rheometer using a 25 mm sandblasted parallel plate geometry. Frequency sweeps were conducted at 0.5% strain from 100 to 0.1 rad/s, and an amplitude sweep was conducted from 0.01 to 100% shear strain at 10 rad/s to verify that all measurements were in the linear viscoelastic regime. Gels for rheology experiments were cast in a mold with a thickness of 1.5 mm. The gap height in all of the rheology experiments was set to 1.2 mm.

### Diffusion Characterization and Analysis

#### Diffusion Experiment Setup

Disposable NMR tubes were filled with approximately 700 μL of the gel solution. Within 24 h of gelation, 350 μL of the NP solution was deposited on top of the gels. In a given batch, 6 NMR tubes with the same gel solution were prepared. One replicate of each of the 6 samples with the same higher-MW ligand but different short:long ligand ratios was measured on each of the three gel mesh sizes, resulting in a total of 18 distinct samples examining the impacts of a grafting density of 1 kDa PEGSH and 18 distinct samples examining the impacts of a grafting density of 2 kDa PEGSH. Each batch of these 36 sample types was repeated 3 independent times for a total of 3 replicates of each NP and gel combination.

NMR tubes were placed in a homemade NMR tube holder during gelation and all measurements. After NP addition, the samples and holder were placed in front of a light pad in an optics bay with no other light sources. A CMOS camera and a home-built Labview code were used to take one image of the samples every hour for 70 h resulting in 70 total time-stamped images.

#### Diffusion Experiment Data Processing

The diffusion experiment and data analysis were conducted following our previously reported protocol.^48^ Briefly, images labeled with timestamps were processed by using a custom Matlab code. Linescans through the interface of the NP and gel were taken for each image. Linescans were fit to two single-sided error functions, as defined by Herriarachi^53^
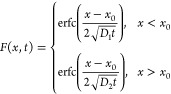
1where *F* is the intensity as a function of time, *D*_1_ is the NP diffusion coefficient within the NP solution, *D*_2_ is the diffusion coefficient within the gel, *t* is time, *x* is the distance from the interface of the gel and NP solution, and *x*_0_ is the initial interface position. The linescan from the first image was used to determine the initial position of the interface between the NPs and gel, and *x*_0_ was held constant in all subsequent fits. For each sample, diffusion coefficients from 20 to 70 h were averaged to give the sample’s average diffusion coefficient.

## Results

### Nanoparticle Characterization

To investigate the effect of ligand density on NP diffusion, we first synthesized a library of NPs with similar Au cores (diameter approximately 3.8 nm) and ligand shells that contain various ratios of short (254 Da) and long (1 or 2 kDa) PEGSH ligands, as summarized in Table 1. We note that in order to investigate the relationship between ligand density and NP diffusion, it was critical to make and rigorously characterize stable NPs with diverse, reproducible ligand densities. Synthesizing NPs with low ligand densities is inherently challenging because having some ligand coverage is critical for achieving colloidal stability. Ligands not only change the particle size but also passivate the NP surface, which both lowers the free energy of the surface and provides a steric barrier to NP coalescence.^44,45^ As a result, colloidally stable NPs with low ligand densities cannot be synthesized by simply reducing the amount of the high-MW ligand bound to the surface. To address this problem, and to better reflect the mixed ligand shells present in many real-world applications, we introduced a short stabilizing ligand (PEGSH, MW = 254 Da), which was co-loaded with our higher-MW ligands of interest. Importantly, this stabilizing ligand was a short oligomer of the same polymer used in the higher-MW ligands, which avoids the introduction of new chemical interactions either with the gel or within the ligand shell. Varying the ratios of the high-MW and short stabilizing ligands used in the NP functionalization then allowed the synthesis of NPs with a wide range of high-MW ligand grafting densities, with short stabilizing ligands occupying the remaining surface sites and providing colloidal stability.

**Table 1:** Sizes and Grafting Densities of Synthesized Nanoparticles

AuNP	ligand density (ligands/NP)^a^	PEGSH MW = 254 Da ligand density (ligands/NP)^a^	hydrodynamic diameter (nm)^b^	core diameter (nm)^c^	ligand shell thickness (nm)^d^	effective grafting density (chains/nm^2^)
	1 kDa					
1A	99 ± 23	0	12.04	3.7 ± 0.6	4.2	2.3
1B	91 ± 26	24 ± 7	10.74	3.6 ± 0.7	3.6	2.2
1C	57 ± 15	46 ± 12	10.96	3.6 ± 0.6	3.7	1.4
1D	8.8 ± 1.6	84 ± 15	10.56	3.7 ± 0.4	3.4	0.21
1E	2.9 ± 0.61	120 ± 25	8.87	3.8 ± 0.5	2.5	0.064
1F	0.72 ± 0.13	79 ± 14	8.43	3.9 ± 0.5	2.3	0.015
						
	2 kDa					
2A	56 ± 10	0	14.60	3.4 ± 0.4	5.6	1.5
2B	52 ± 11	17 ± 4	14.72	3.5 ± 0.5	5.6	1.4
2C	31 ± 5	66 ± 11	13.69	3.6 ± 0.4	5.0	0.76
2D	5.1 ± 0.9	67 ± 12	11.57	3.4 ± 0.4	4.1	0.14
2E	2.0 ± 0.4	85 ± 17	9.15	3.7 ± 0.5	2.7	0.047
2F	0.59 ± 0.1	51 ± 9.3	7.79	3.3 ± 0.4	2.2	0.017

a Determined via NMR, ICP, and TEM of samples.

b Determined via DLS of samples.

c Determined via TEM of samples (TEM micrographs are shown in Figure S2).

d Calculated by subtracting core size from hydrodynamic diameter and dividing by 2.

Because the short and long ligands may not bind equally to the NP surface, however, it was critical to characterize the composition of the ligand shell that formed on the NPs rather than assuming that it was equal to the mixture of ligands used in the functionalization step. To this end, we quantified the ligand shell compositions using NMR and ICP-OES following a modification of a previously reported protocol.^52^ This analysis showed that functionalization with mixtures of short and long ligands successfully varied the loading of the longer ligands from 0 to 100 chains per NP (corresponding to grafting densities of 0–2.3 chains/nm^2^) for the 1 kDa system and 0 to 60 chains per NP (corresponding to grafting densities of 0–1.5 chains/nm^2^) for 2 kDa system (see Table 1). The loading of the short (254 Da) stabilizing ligands correspondingly varied such that the total grafting density of the ligands on each NP was roughly constant. For convenience, however, in the rest of this article, we use the term effective grafting density to refer specifically to the coverage of the higher-MW ligand. We note that 1 and 2 kDa PEGs have radii of gyration on the order of 1 and 1.5 nm,^54,55^ respectively, corresponding to overlap concentrations on the particle surfaces of approximately 0.3 chains/nm^2^ for the 1 kDa ligands and 0.15 chains/nm^2^ for the 2 kDa ligands. As such, the range of effective grafting densities studied encompasses both the semidilute (samples A−C in each series) and dilute (samples D−F in each series) regimes.

**Figure 1:**
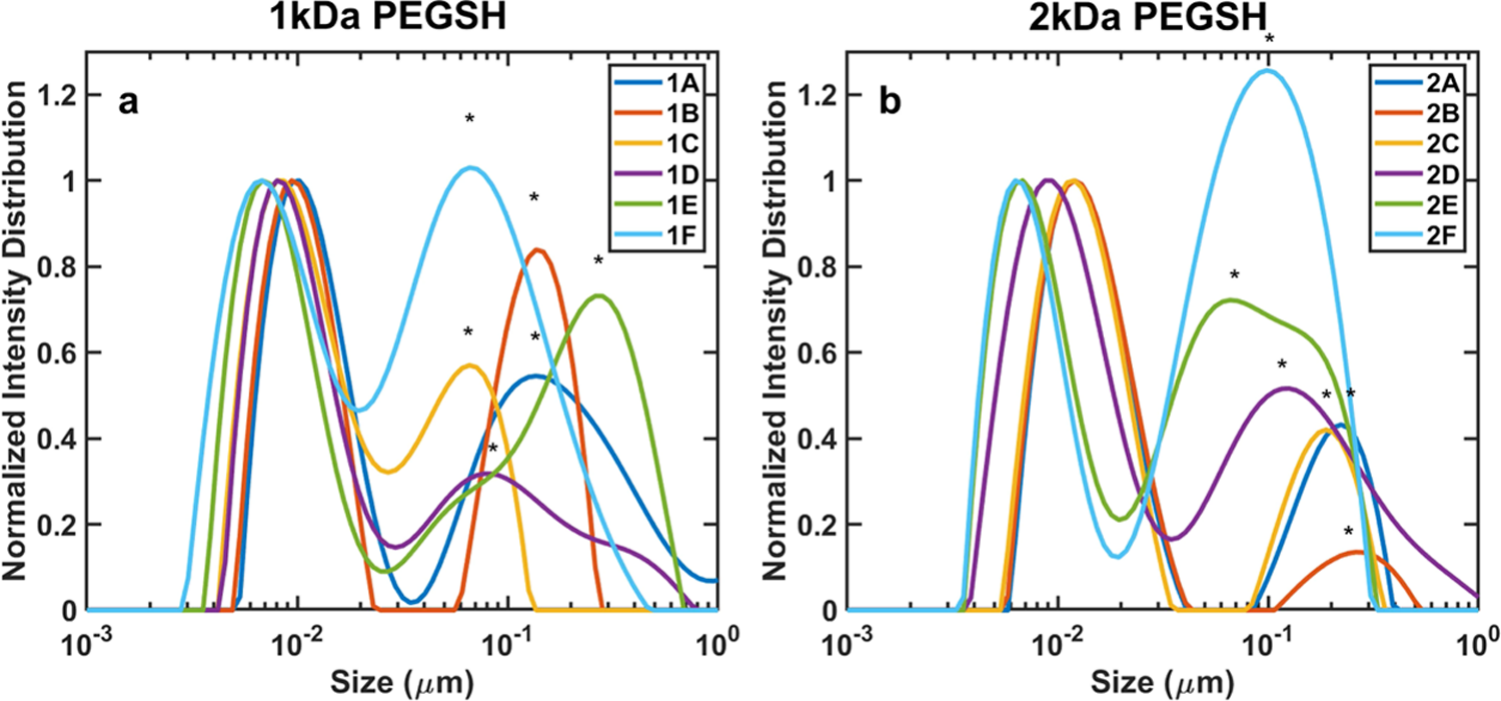
Normalized DLS traces of NPs with (a) 1 kDa PEGSH (b) and 2 kDa PEGSH ligands. Stars indicate peaks of detritus from molecular-weight cutoff filters used to purify the samples (see ref (48)).

The hydrodynamic diameters of the NPs were then measured via DLS, as shown in Figure 1 and summarized in Table 1. As seen in these data, NPs with high effective grafting densities, or tightly packed higher-MW ligand shells (1A and 2A), had larger hydrodynamic diameters than NPs with fewer higher-MW ligands (1F and 2F). The ligand shell thickness, calculated from the difference between the hydrodynamic radius of the NPs and the radius of the Au core, varied from 2 to 4 nm for NPs with 1 kDa ligands and from 2 to 6 nm for NPs with 2 kDa ligands, with the ligand shell thickness increasing with the effective grafting density. We note that the contour lengths of the 254 Da PEGSH, 1 kDa PEGSH, and 2 kDa PEGSH are approximately 1.5, 6, and 12 nm, respectively. The calculated ligand shell thicknesses thus indicate that the ligands are not fully extended on the surface of the NPs, even in the samples with the highest effective grafting densities. The relative ligand shell thicknesses for the 1A and 2A samples were consistent, however, with the scaling predicted by the Daoud–Cotton model for spherical brushes in the semidilute regime.^56,57^

### Gel Mesh Size Characterization

Gels with three different average mesh sizes were synthesized by free-radical polymerization of acrylamide/bis(acrylamide) mixtures, as summarized in Table 2. The acrylamide concentration was held constant at 3 w/v %, while the bis(acrylamide) cross-linker concentration was varied between 0.06 and 0.0226 w/v %. After gelation, the zero-frequency storage modulus of the gels was determined via small-amplitude oscillatory shear rheology. This modulus was then used to estimate the average mesh size of the gels using the elastic blob theory,^58^ which was previously established as the best estimate of the mesh size for these swollen networks.^48^ Briefly, this model assumes that the mesh size is equal to the size of the elastically effective chains, ξ
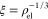
2where ρ_el_ is the number density of elastic blobs and is calculated from the zero-frequency shear modulus (*G*_(0)_^′^) via
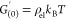
3where *T* is temperature and *k*_B_ is Boltzmann’s constant.^58^ This analysis revealed that the synthesized gels had average mesh sizes between 35 and 62 nm, consistent with our prior work.^48^

**Table 2:** Moduli and Average Mesh Sizes of Polyacrylamide Gels

acrylamide concentration (w/v %)	bis-acrylamide concentration (w/v %)	zero-frequency storage modulus *G*_(0)_^′^^b^ (Pa)	average gel mesh size (nm)^a^
3	0.0600	94.4	35
3	0.0400	44.2	45
3	0.0226	17.1	62

a Average gel mesh size was estimated via the elastic blob model.

b Frequency sweeps of gels and linear fits of data to determine (*G*_(0)_^′^) can be found in SI Figure S6.

### Diffusion Experiment Results

**Figure 2:**
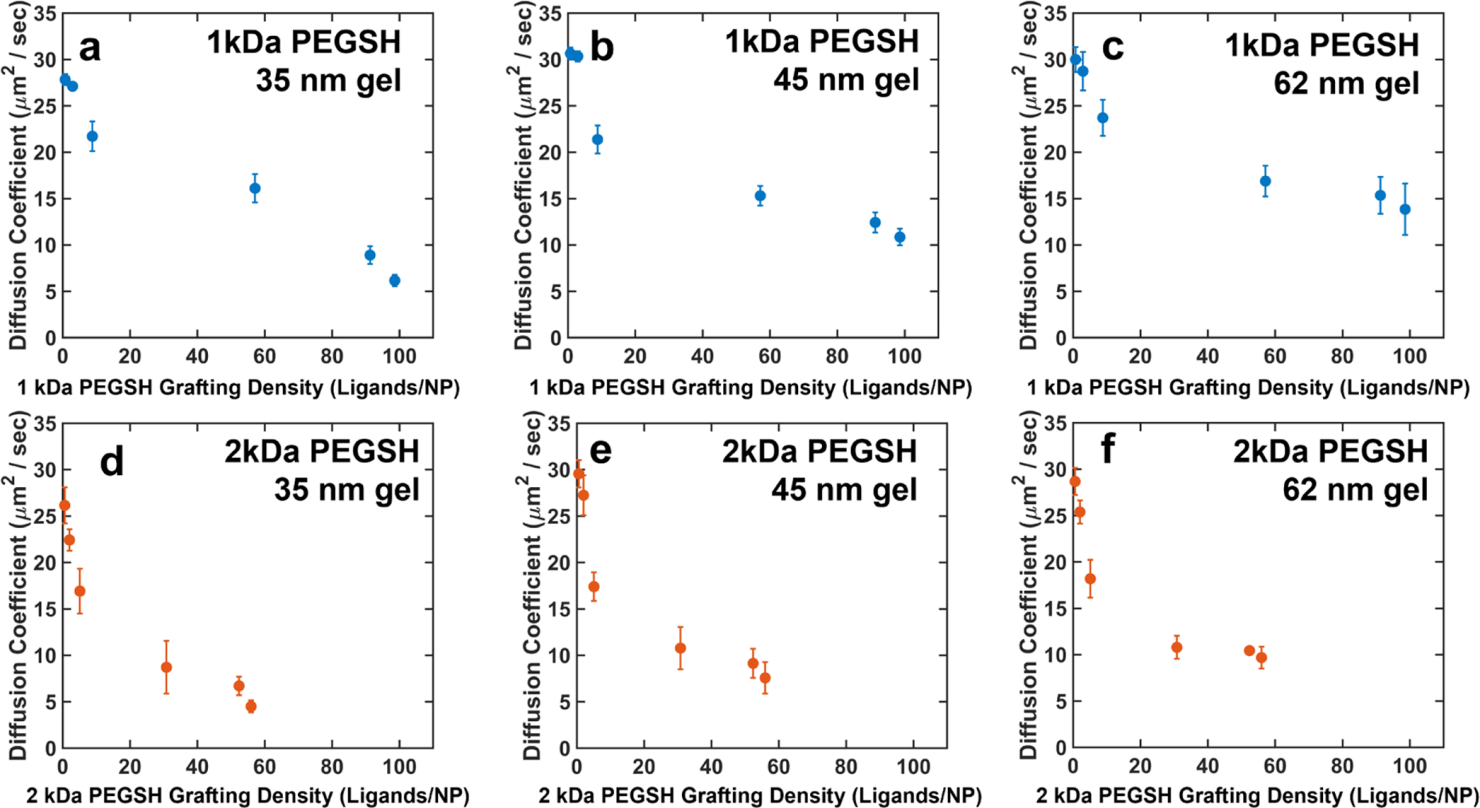
Diffusion coefficients for NPs with varying effective grafting densities of (a–c) 1 kDa and (d−f) 2 kDa ligands in gels with average mesh sizes of (a, d) 35 nm, (b, e) 45, and (c, f) 62 nm. Error bars represent the standard deviation in the diffusion coefficient calculated from three independent trials.

Diffusion experiments were carried out for each of the 12 NP types on each of the three gels following the protocol described above. The resulting diffusion coefficients are plotted as a function of effective grafting density in Figure 2. As seen in these data, for each ligand/gel combination, the diffusion coefficients of the NPs generally decreased as the effective grafting density increased. NPs functionalized with high densities of 1 kDa ligands had diffusion coefficients slightly faster than those of the NPs functionalized with 2 kDa ligands, as would be expected from their relative hydrodynamic diameters. However, the NPs with the lowest coverages of the higher-MW ligands had approximately the same diffusion coefficients for both the 1 and the 2 kDa systems. For both the 1 and 2 kDa systems, the diffusion coefficients of the particles in the gels were all less than half that of the particles in free solution (see the Supporting Information), indicating that interactions with the gel do mediate nanoparticle diffusion.

**Figure 3:**
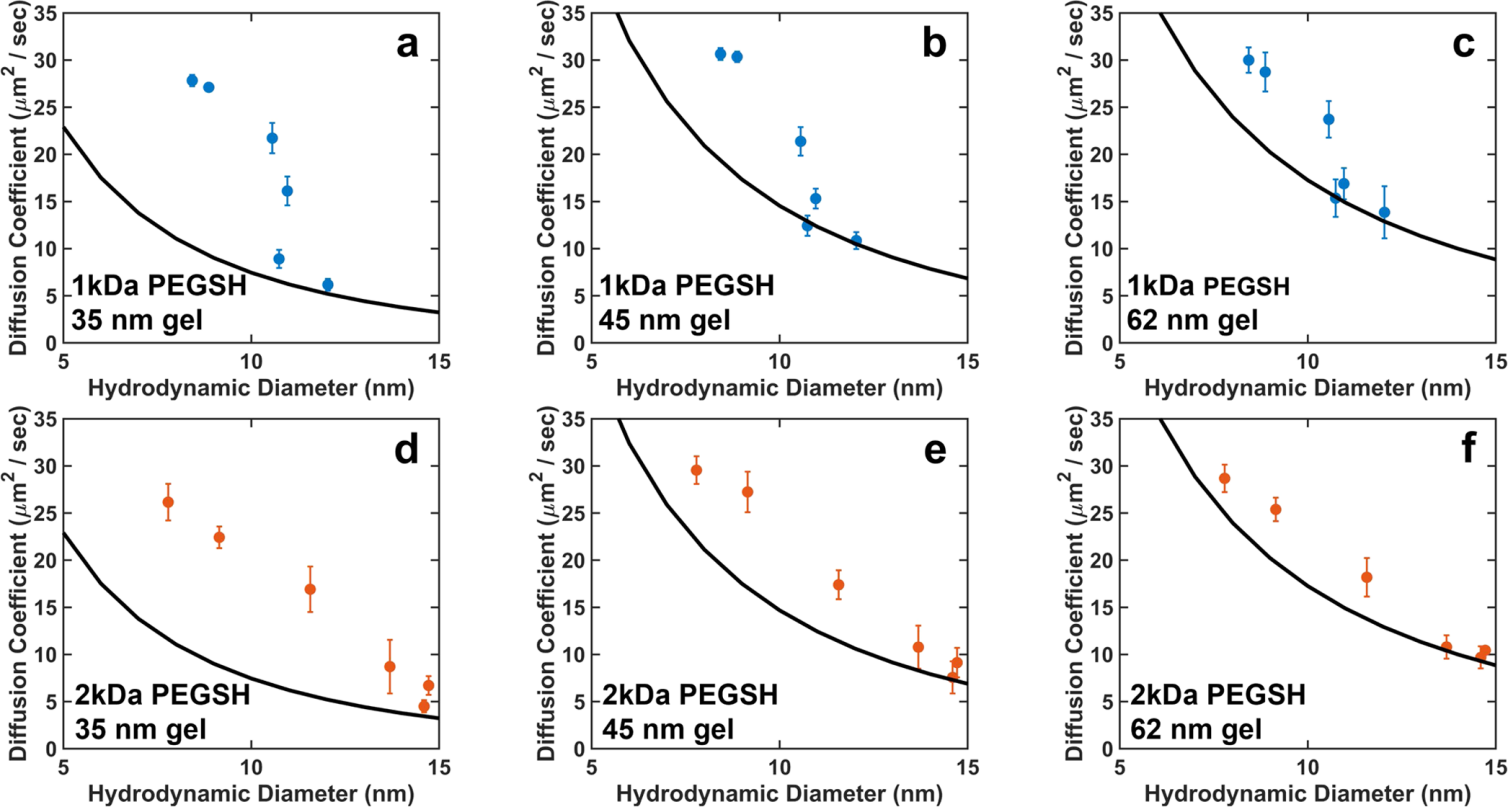
Diffusion coefficient as a function of hydrodynamic diameter for NPs with (a–c) 1 kDa and (d−f) 2 kDa ligands in gels with average mesh sizes of (a, d) 35 nm, (b, e) 45, and (c, f) 62 nm. The black line on each plot represents the diffusion coefficient predicted from fits to our prior data on densely grafted particles (see the Supporting Information and ref (48)). Error bars represent the standard deviation in the diffusion coefficient calculated from three independent trials.

Plotting the same diffusion coefficients as a function of the hydrodynamic diameter of the particles measured in aqueous solution (Figure 3) reveals that the diffusion coefficient decreases with increasing hydrodynamic diameter, as expected from our previous work.^48^ To determine if the changes in diffusion coefficient resulted from changes in hydrodynamic diameter alone, the measured diffusion coefficients were compared to the diffusion coefficients predicted for densely grafted particles with the same hydrodynamic radii (Supporting Information). As seen in Figure 3, NPs with high effective grafting densities gave diffusion coefficients consistent with this model. However, NPs with low effective grafting densities gave diffusion coefficients that were significantly higher than those predicted from their hydrodynamic diameters. These results indicate that NPs with low effective grafting densities (i.e., NPs that are functionalized mostly with short ligands) behave differently in the gels than particles with high effective grafting densities (i.e., NPs that are functionalized mostly with long ligands), even when their hydrodynamic diameters are the same. We note that the diffusion coefficients of the smallest particles are mostly insensitive to the average mesh size of the gels and only about half that of the particles in pure water, which could indicate that the local viscosity of the gels is higher than in aqueous solution. Because the gels are mostly water (ϕ_pol_ ≈ 0.025) and acrylamide–PEG interactions are generally weak,^59^ however, the hydrodynamic diameters of the particles in the gels are expected to be similar to those measured in free solution.

**Figure 4:**
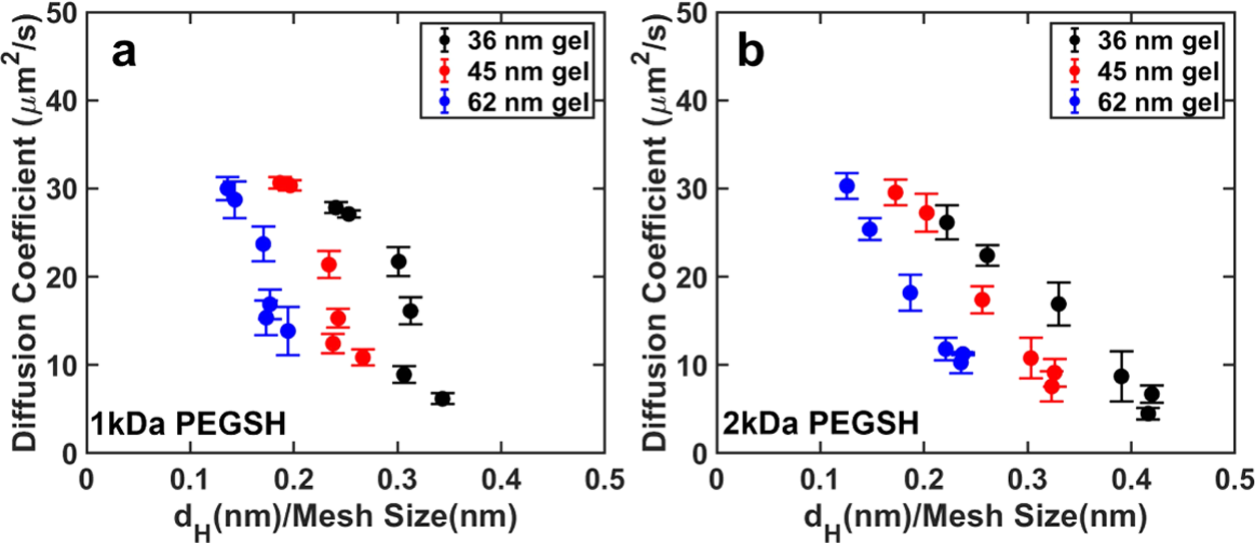
Diffusion coefficient as a function of confinement ratio (hydrodynamic diameter/average mesh size) for NPs with (a) 1 and (b) 2 kDa ligands across all three mesh sizes of gels. Error bars represent the standard deviation in the diffusion coefficient calculated from three independent trials.

If the hydrodynamic diameter was the primary driver of the NP diffusion behavior, we would expect the diffusion coefficients to be well-predicted by the confinement ratio of the NPs in the gels, where the confinement ratio of a particle is equal to its hydrodynamic diameter divided by the gel’s average mesh size. Indeed, for NPs with high ligand densities, the diffusion coefficients of NPs in different gels were previously all found to collapse onto a single curve when plotted against this quantity.^48^ By contrast, as shown in Figure 4, this relationship did not hold for the NPs with mixed ligand shells investigated in this work. Within each series of gels with the same average mesh size, the diffusion coefficients did decrease with an increase in confinement but the confinement ratio was not a good predictor of the diffusion coefficient across gel mesh sizes. This deviation again illustrates that the behavior of NPs with low effective grafting densities is distinct from that of NPs with high grafting densities, even for NPs with the same hydrodynamic diameter.

**Figure 5:**
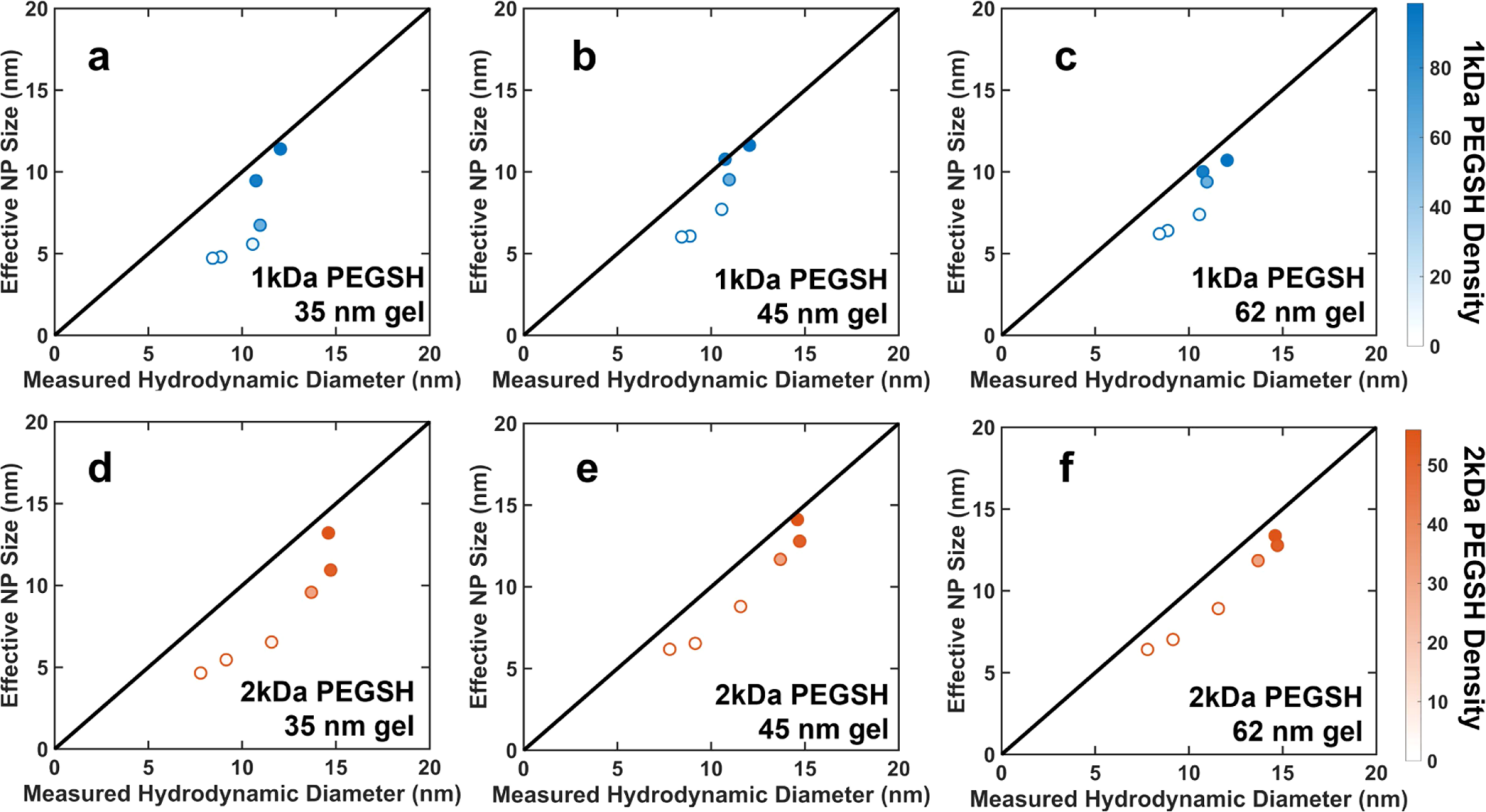
Effective NP size in the gel environment as a function of hydrodynamic diameter for NPs with (a–c) 1 kDa and (d−f) 2 kDa ligands in gels with average mesh sizes of (a, d) 35 nm, (b, e) 45, and (c, f) 62 nm. The color of the markers indicates the effective grafting density of the higher-MW ligand, in average number of chains/particle, and the black line indicates the points at which the measured hydrodynamic diameter is equal to the effective NP size.

Finally, to better illustrate the differences between the diffusion mechanisms of the NPs with different effective grafting densities, we used the fits to data on densely grafted particles, shown in Figure 3 and described in the Supporting Information, to estimate the effective size of the NPs in the gel. The effective NP size was defined as the particle size that the fits predicted would give the experimentally measured diffusion coefficient. The effective NP size is plotted as a function of the NP hydrodynamic diameter, as measured via DLS, in Figure 5. In this figure, the color gradient of the markers reflects the grafting density of the higher-MW ligands, with the darkest markers indicating NPs with the highest effective grafting densities and the lightest markers indicating NPs with the lowest effective grafting densities. The black line indicates the points at which the hydrodynamic diameters of the NPs measured in free solution and their effective sizes in the gel are equal. For the NPs with 1 kDa ligands, the two samples with the highest effective grafting densities (as indicated by the darkest markers) fall on this line, indicating that their effective sizes in the gel are roughly equal to their measured hydrodynamic diameters. The remaining samples, which have lower effective grafting densities, fall below the line, indicating that they diffused through the gel as if they were smaller than indicated by their hydrodynamic diameters. The NPs with the lowest effective grafting densities plateau at an effective diameter of approximately 5 nm, which is just above the diameter of the NP cores. The NPs with 2 kDa ligands exhibited similar trends. These results, and particularly the deviation of the effective sizes of the NPs with low effective grafting densities from the predictions for densely grafted particles, again suggest that the mechanism of diffusion is different for the loosely grafted NPs than it is for the densely grafted NPs, as described in more detail, below.

## Discussion

In this work, we investigated the effect of ligand density on the diffusion of NPs in hydrogel nanocomposites. In our previous work on NPs with high ligand densities, we found that the diffusion coefficients were independent of the relative sizes of the hard cores and softer ligand shells and were instead well-predicted by the NPs’ hydrodynamic diameters alone. This observation led us to conclude that the ligand shell behaved as a rigid layer surrounding the NP core. We hypothesized that this behavior arose because the ligand shells were densely packed, and that reducing the ligand shell density might allow the ligand shell to behave as a softer, more flexible material that would facilitate faster diffusion through confined spaces.^60,61^

Here, we tested this hypothesis by using mixtures of short and long ligands to vary the effective grafting density of the ligand shell and characterized the diffusion of the resulting NPs through cross-linked polyacrylamide hydrogels. Measurement of the diffusion coefficients of these NPs in polyacrylamide gels revealed that the ligand shell density did indeed impact the diffusion mechanism. Decreasing the effective grafting density by decreasing the number of higher-MW ligands per NP decreased the hydrodynamic diameters of the NPs, which resulted in an increase in their diffusion coefficients. However, the increases in the diffusion coefficients were more pronounced than expected from the changes in the hydrodynamic diameter alone. When compared to the diffusion coefficients predicted from their hydrodynamic diameters, the diffusion of NPs with low effective grafting densities was significantly faster than the diffusion of NPs with high effective grafting densities with the same hydrodynamic diameter. Additionally, the confinement ratio of the NPs was not a good predictor of their diffusion coefficients. Taken together, these results indicate that the NPs with low effective grafting densities had a smaller effective size in the gel than NPs with high effective grafting densities even when they had the same size in free solution, and suggest that reducing the grafting density of the higher-MW ligand fundamentally changes the mechanism of NP diffusion.

Previous measurements of the diffusion of NPs in the high grafting density limit yielded bulk diffusion coefficients that appeared to be well-described by Cai and Rubinstein’s hopping model.^31^ While there are some limitations to applying this model in the present system, as discussed in more detail below, it provides a useful framework for conceptualizing how ligand density might impact the diffusion process. In the hopping model, the NP is modeled as a solid spherical particle and the gel as a network of fluctuating chains. The diffusion of NPs with sizes on the order of the mesh size is dictated by the time scale on which the network strands fluctuate enough to stretch around the NPs and allow them to hop from one location to the next. For particles with densely grafted ligand shells that behave as solid spheres, diffusion is expected to depend on the time scale of these environmental fluctuations. By contrast, if the ligand shells of NPs with low effective grafting densities are able to fluctuate on experimentally relevant time scales, they may not behave as solid spheres and may instead be able to “fit” through smaller spaces in the gel, facilitating faster diffusion.

Theoretical and computational work on polymer-grafted NPs provides useful insight into the potential role of the relaxation of the grafted chains.^49,50,62^ Theoretical models of polymer-grafted NPs in polymer melts predict that at low grafting densities the grafted chains relax independently of one another, and the effective diameter of the NPs in a melt of other polymer chains is close to their core size. At high grafted chain densities, on the other hand, the same models predict that the grafted chains are hydrodynamically coupled, and the effective diameter of the NPs in the melt is that of the core plus the thickness of the nondraining ligand shell.^49^ Similar effects have also been observed in both coarse-grained and atomistic molecular dynamics simulations.^50,62^ Of particular relevance to the present work, atomistic simulations of PEG-grafted nanoparticles in water suggest that at grafting densities below the overlap concentration, the end-to-end distance of the chains fluctuates quickly, with 500 Da PEG chains undergoing end-to-end distance changes of up to 1 nm on time scales of less than 5 ns,^62^ much faster than the μs–ms relaxations reported for polymer chains in acrylamide gels.^63^ At grafting densities well above the overlap concentration, on the other hand, the same chains were effectively locked in place, with the end-to-end distances varying by only about 0.2 nm over the entire production run.^62^

In the context of these models and simulations, we interpret our experimental data as follows. At high effective grafting densities, the ligands are hydrodynamically coupled, and their conformational fluctuations are significantly restricted. As a result, it is faster for the network strands to stretch around the entirety of the ligand shell than it is for the ligand shell to contract, and the diffusion time scale is dictated by the fluctuations of the network strands. However, as the effective grafting density and packing of the higher-MW ligands decrease, these chains experience a less-hindered environment, allowing for faster and larger conformational fluctuations. As a result, it becomes faster for the higher-MW ligands to retract and allow the NPs to fit through an available space in the gel without waiting for the matrix chains to relax. The faster relaxation dynamics of the NP ligands leads to an effectively smaller “hard” surface and faster diffusion coefficients, even when the hydrodynamic diameters of the NPs (which are more dependent on the relative relaxation times of the ligand shell and the highly mobile solvent) remain high. This transition between the network-dominated and ligand-dominated regimes should occur when the ligand shell transitions from the semidilute brush regime, where fluctuation in the ligand shell thickness is restricted by the crowding of neighboring chains, to the dilute regime, where ligand fluctuations are independent and larger in amplitude; this prediction is qualitatively consistent with the change in diffusion behavior observed in this work, as the effective grafting densities decrease from above the overlap concentration to below the overlap concentration on the NP surface.

While the hopping model serves as a useful conceptual framework for understanding how ligand fluctuations affect nanoparticles’ interactions with their environment, we note that there are a number of limitations to directly applying the hopping model to the present system. First, while our previous measurements of the diffusion coefficients of densely grafted particles appeared to be consistent with hopping model predictions (and this model is correspondingly used to estimate the diffusion coefficients of particles in the densely grafted limit in this work), strong conclusions cannot be drawn about the diffusion mechanism without the observation of individual hopping events, which are not possible in the bulk diffusion measurements reported here. Second, hopping diffusion is only strictly expected to occur when the particle size is larger than the gel mesh size, while in the present work, the hydrodynamic diameters of the nanoparticles are all smaller than the average mesh sizes of the gels. Because the chains between cross-links are unlikely to be fully extended and thus still fill some of the space between the cross-links, we suspect that particles smaller than the local distance between cross-links may still be transiently trapped by the network chains and require thermal fluctuations of these chains to generate large enough openings for the particle to move. Gels prepared by free-radical polymerization are, however, also heterogeneous, with significant spatial variation in both the cross-link density and the mesh size.^64^ As such, the “mesh size” obtained from rheology experiments may not accurately reflect the local mesh experienced by the majority of particles in the system. Recently, for example, Rose et al. showed that nanoparticles in tetra-PEG gels remained nearly immobile when the particle size was comparable to the average gel mesh size. As the defect content of the gels (and thus their average mesh sizes) increased, the particles split into distinct “immobile” and “mobile” populations as they explored regions of higher or lower cross-link density, but a substantial fraction of the particles remained immobile even at confinement ratios as low as 0.2.^65^ The bulk diffusion coefficients measured here likely reflect an average of the high- and low-mobility NP populations, but it is possible that particles smaller than the average gel mesh size may undergo hopping diffusion within the more densely cross-linked regions of the gel and that this is reflected in the bulk diffusion measurements if the majority of the particles explore these regions. Regardless of the specific local diffusion mechanism, however, the much lower diffusion coefficients measured for particles in gels than in free solution indicate that the gels provide physical barriers that limit the diffusion of the NPs, and fluctuations of the ligand shell conformation appear to play an important role in enabling the NPs to move around these barriers.

Together, this work demonstrates that ligand shell density and the resulting changes in ligand shell relaxation play an important role in NP diffusion in confined environments. In particular, as the grafting density of the higher-MW ligands decreases, there appears to be a change in the mechanism of diffusion, as the ligand shell fluctuations become fast enough and large enough to facilitate movement through the gel. This work provides important insights into how mixed ligand shells (which are required for many real-world applications) may affect the diffusion of NPs and suggests a number of possibilities for future work. First, the development of theoretical models that account for the relative time scales and amplitudes of conformational fluctuations of the grafted chains on the NPs and the surrounding matrix will be important for predicting where transitions in diffusion behavior occur in these systems. From an experimental perspective, rigorous characterization of the NP ligand shell is also critical for understanding these trends, and quantitative techniques for determining the number of grafted chains can and should be employed whenever predicting or interpreting NP diffusion in complex environments. Second, as noted above, the gels investigated here are randomly cross-linked and may exhibit substantial heterogeneity in both the spatial distribution of cross-links and the network strand lengths.^64^ Future experiments on more homogeneous systems, such as tetra-PEG gels,^58^ may provide further physical insight into the role of network strand fluctuation in these systems. Finally, we also note that in the present experiments, the ligand shells were selected to have minimal chemical interactions with the gel,^48^ facilitating the interpretation of the results solely in terms of how the distribution of ligand lengths and resulting changes in ligand shell packing affect the physics of the diffusion process. However, in real-world systems, non-negligible chemical interactions are often present. Future experiments in which the polymer chemistries are changed to systematically introduce different chemical interactions between the NPs and the matrix will help to develop models that more comprehensively account for the different factors that dictate NP diffusion under confinement. Taken together, these efforts promise to both advance the fundamental understanding of the physics of NP–gel composites and advance the development of biological therapeutics and other technologies relying on the motion of NPs with complex surface functionalization in crowded environments.

## Conclusions

In this work, the diffusion of PEGylated NPs in polyacrylamide hydrogels was studied as a function of the ligand density and gel mesh size. Colloidally stable NPs with varying densities of higher-molecular-weight ligands were prepared by co-loading mixtures of higher-molecular-weight ligands and short stabilizing ligands, yielding NPs with as many as 100 high-MW ligands/NP to as few as 1–2 ligands/NP. As the grafting density of the higher-MW ligand decreased, the diffusion coefficient of the NPs increased. Interestingly, however, the increase in diffusion coefficient was larger than expected from the change in the NPs’ hydrodynamic diameters alone, suggesting that the relaxation of the ligand chains plays an important role in facilitating the diffusion of NPs in crowded environments. This result suggests that treating NPs as hard spheres misses important aspects of the NP–gel interactions and theoretical models of NP diffusion should be extended to account for the characteristics of the ligand shell. More broadly, we hope that this work will not only inform models for predicting the diffusion of NPs in polymer networks but also improve the understanding of the features that can be used to tailor NP behavior in complex environments such as biological materials and inform the design of NP-based therapeutics and other functional hydrogel nanocomposite materials.

## Supporting Information Available

Characterization data for nanoparticles and gels; details of fits used to analyze data (PDF)
