# Supplementary material for: Role of Ligand Shell Density in the Diffusive Behavior of Nanoparticles in Hydrogels
Source: J Phys Chem B. 2023 Oct 19;127(43):9366–77. doi: 10.1021/acs.jpcb.3c03249 (PMC10626584; doi:10.1021/acs.jpcb.3c03249)
Supplement: Supplementary file 1 — jp3c03249_si_001.pdf [file jp3c03249_si_001.pdf]

Supporting Information: The role of ligand shell density in the diffusive behavior of nanoparticles in hydrogels

Paige J. Moncure, Jill E. Millstone, and Jennifer E. Laaser*

Department of Chemistry, University of Pittsburgh, Pittsburgh, PA, 15260

E-mail: j.laaser@pitt.edu

Phone: (412)383-0125

Contents

Ligand Quantification **S-3**

Figure S1 - NMR spectra of 254 Da PEGSH, 1 kDa PEGSH, and 2 kDa PEGSH. S-3

Figure S2 - Standard curves for NMR ligand quantification. S-4

Figure S3 - NMR spectra of 1kDa PEGSH samples. S-5

Figure S4 - NMR spectra of 2kDa PEGSH samples. S-6

Table S1 - NMR integration and ICP data. S-6

Table S2 - 1kDa ligands added vs ligands on particle. S-7

Table S3 - 2kDa ligands added vs ligands on particle. S-7

TEM Characterization **S-8**

Figure S5 - TEM images of the 1kDa PEGSH capped NPs. S-8

Figure S6 - TEM images of the 2kDa PEGSH capped NPs. S-9

Gel Characterization **S-10**

Figure S7 - Rheology of hydrogels. S-10

Comparison of Diffusion in Gels vs. Free Solution **S-11**

Figure S8 - Comparison to Diffusion in Free Solution. S-11

Diffusion Coefficients of Densely-Grafted Particles **S-12**

Figure S9 - Fit of ξ^2/τ_0 vs. mesh size used to obtain hopping model parameters. . S-13

Table S4 - Interpolated fit variables for the hopping model. S-13

References **S-14**

Ligand Quantification

As described in the main text, the ligand coverage of each nanoparticle was determined using a combination of ICP-OES and quantitative NMR. In the NMR experiments, spectra of digested NP samples were analyzed to determine the absolute concentration of each ligand type in the digested sample. These concentrations were determined as follows. First, spectra were obtained for solutions containing known concentrations of a single ligand and a known concentration of acetonitrile (ACN) as an internal standard. Representative spectra of these standard solutions for each ligand, and the peaks used in the NMR analysis, are shown in Figure S1.

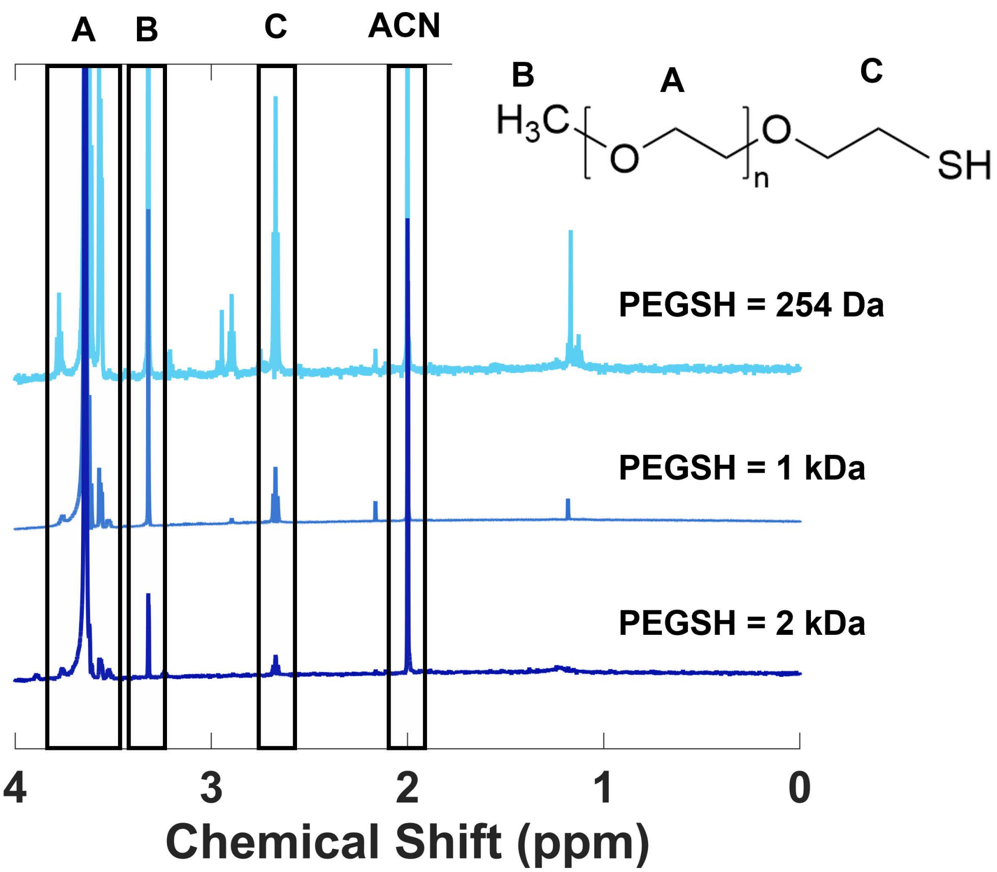

Figure S1: NMR spectra of 0.5 M solutions of 254 Da PEGSH, 1 kDa PEGSH, and 2 kDa PEGSH with 5 μL 0.24% v/v ACN. Spectra are normalized to the A peak at 3.65 ppm.

The integrals of the “A” and “B” peaks, representing the intensities of the signals from

the PEG backbones and the methyl end groups, respectively, were then normalized to the ACN internal standard and used to construct four standard curves, shown in Figure S2.

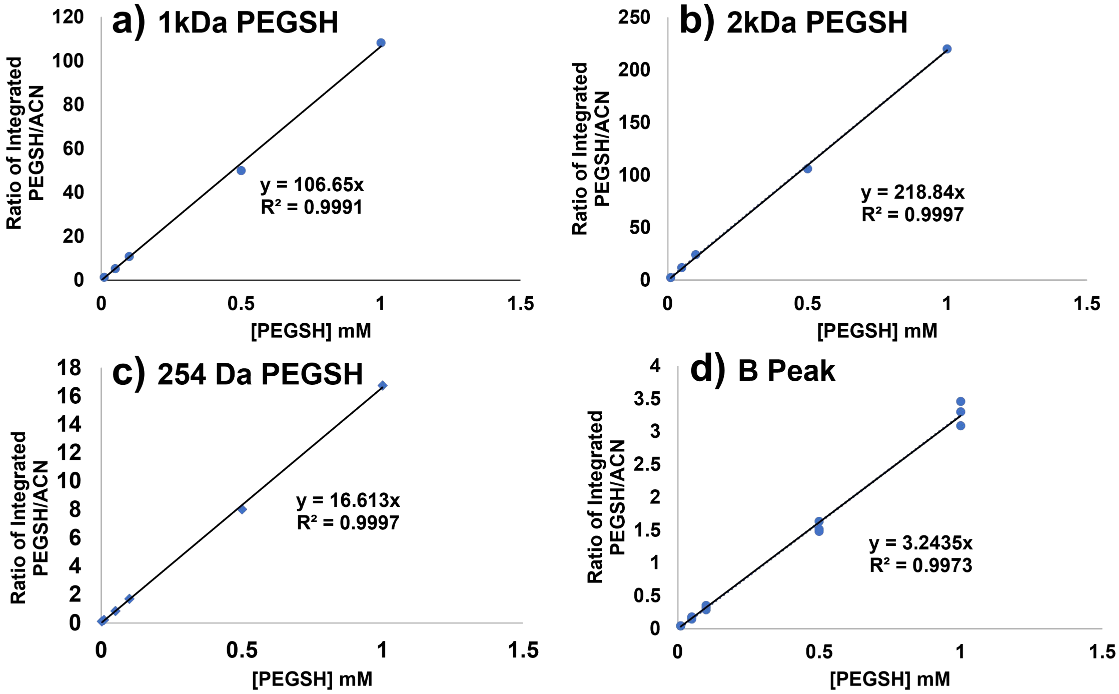

Figure S2: Standard curves for the backbone ("A") peaks for solutions of (a) 1 kDa PEGSH, (b) 2 kDa PEGSH, (c) 254 Da PEGSH, and (d) standard curve for the end-group ("B") peak for all three ligand types. The integrals of the peaks of interest were divided by the integral of the ACN internal standard peak for all standard curves.

The spectra of digested samples containing mixed high-MW and 254 Da PEGSH ligands (Figs. S3 and S4) were then analyzed as follows. First, as with the standard solutions, the integrals of the "A" (backbone) and "B" (end-group) peaks were each divided by the integral of the acetonitrile internal standard. The integral of the B peak was then compared to the standard curve for this peak to determine the total concentration of ligands (C_{tot}) in the sample. Finally, the concentrations of the low- and high-MW ligands were determined from the integral of the A peak (I_A) by solving the following system of equations

$$I_A = A_{low}c_{low} + A_{high}c_{high} \tag{1}$$

$$c_{tot} = c_{low} + c_{high} \tag{2}$$

where A_{low} and A_{high} are the slopes of the calibration curves for the low- and high-molecular weight ligands, respectively, and c_{low} and c_{high} are their concentrations in the mixed sample. The peak intensities from the spectra in Figs. S3 and S4 that were used to carry out this analysis are summarized in Table S1; the resulting concentrations were then combined with the Au concentrations from ICP-OES (also summarized in Table S1) and the NP core sizes from TEM (Figs. S5 and S6) to determine the number of each type of ligand per particle as described in the main text.

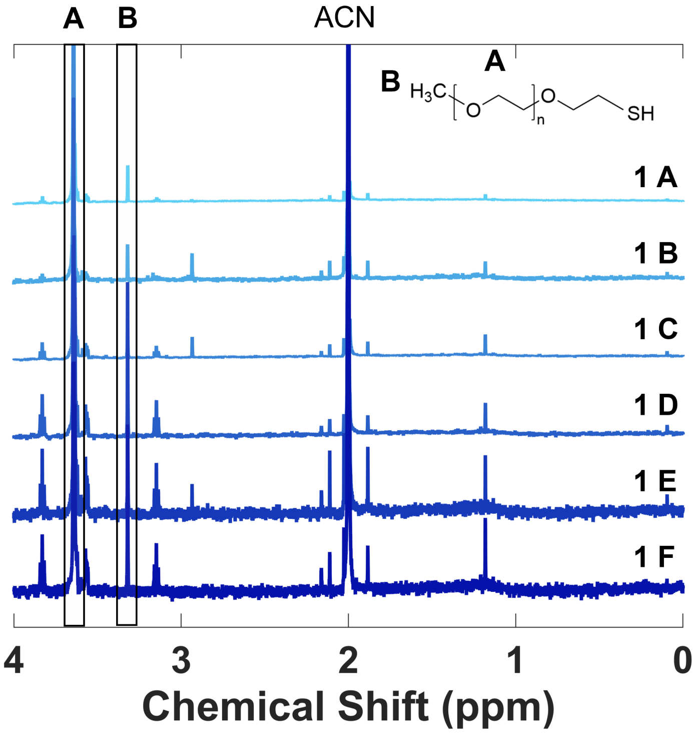

Figure S3: NMR spectra of NP samples functionalized with mixtures of 1 kDa PEGSH and 254 Da PEGSH. All spectra are normalized to the A peak at 3.65 ppm. The peak integrals used in the ligand density calculations are summarized in Table S1.

While our primary goal in carrying out the ligand quantification experiments was to measure the actual ligand density for each set of particles, we note that this analysis also revealed that the ratio of high and low MW ligands that attached to the particle surfaces was somewhat different than that added to the solution in the functionalization step. As shown in Tables S2 and S3, the ratio of high MW ligands to low MW ligands on the particles was

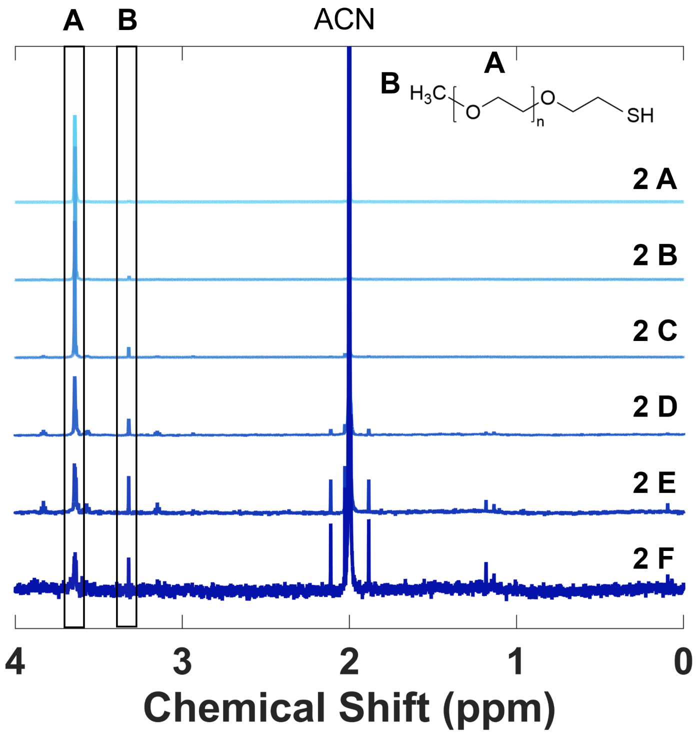

Figure S4: NMR spectra of NP samples functionalized with mixtures of 2 kDa PEGSH and 254 Da PEGSH. All spectra are normalized to the A peak at 3.65 ppm. The peak integrals used in the ligand density calculations are summarized in Table S1.

Table S1: NMR and ICP raw Data

Sample ID	I_A/I_{ACN}	I_B/I_{ACN}	Au Conc. by ICP (ppm)
1A	1.4051	0.0392	2.77
1B	0.3858	0.0138	0.72
1C	0.8140	0.0388	2.24
1D	0.4008	0.0502	3.53
1E	0.1457	0.0245	1.41
1F	0.1403	0.0254	2.43
2A	1.8777	0.0265	2.60
2B	2.732	0.0509	4.04
2C	1.079	0.0422	2.60
2D	0.1459	0.0149	1.04
2E	0.4860	0.0072	0.54
2F	0.0168	0.0028	0.24

generally somewhat lower than the ratio in the mixture used in the functionalization step. This result is not unexpected, as the high MW ligands likely have a harder time packing on the NP surface, but highlights the importance of characterizing the *actual* ligand coverages on the NPs rather than assuming they directly reflect the amounts in solution.

Table S2: 1kDa PEGSH Ligands Added

Sample ID	[PEGSH MW = 1 kDa Ligand Density] (mmol)	[PEGSH MW = 254 Da Ligand Density] (mmol)	[1 kDa PEGSH]: [254 Da PEGSH] Added	[1 kDa PEGSH]: [254 Da PEGSH] Measured
1A	0.095	0	N/A	N/A
1B	0.0425	0.0046	9.2391	3.876
1C	0.0095	0.0061	1.5574	1.225
1D	0.00095	0.0091	0.1044	0.105
1E	0.000095	0.0121	0.0079	0.024
1F	0.000019	0.0137	0.0014	0.009

Table S3: 2kDa PEGSH Ligands Added

Sample ID	[PEGSH MW = 2 kDa Ligand Density] (mmol)	[PEGSH MW = 254 Da Ligand Density] (mmol)	[2 kDa PEGSH]: [254 Da PEGSH] Added	[2 kDa PEGSH]: [254 Da PEGSH] Measured
1A	0.095	0	N/A	N/A
1B	0.0425	0.0046	9.2391	3.084
1C	0.0095	0.0061	1.5574	0.464
1D	0.00095	0.0091	0.1044	0.076
1E	0.000095	0.0121	0.0079	0.024
1F	0.000019	0.0137	0.0014	0.012

TEM Characterization

Representative TEM images of the 1kDa PEGSH and 2kDa PEGSH capped NPs are shown in Figures S5 and S6. The size distributions of the NP cores are shown in the inset histograms, and the core sizes reported in the main text were determined by averaging the diameters of 200 NPs.

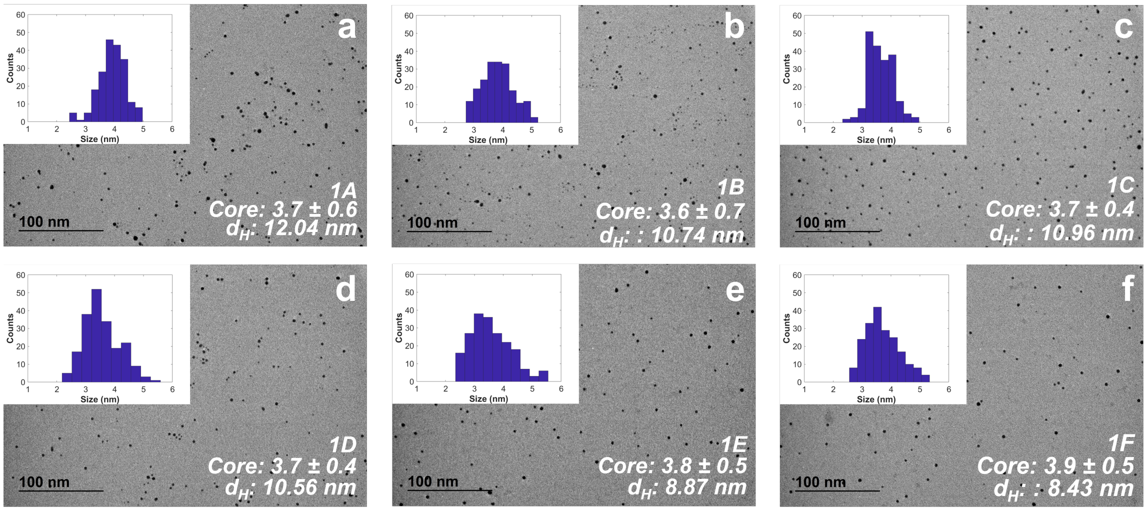

Figure S5: Representative transmission electron micrographs of 1kDa PEGSH NP samples 1A, 1B, 1C, 1D, 1E, and 1F. Histograms of core size distribution are included in the upper left hand corner of each image.

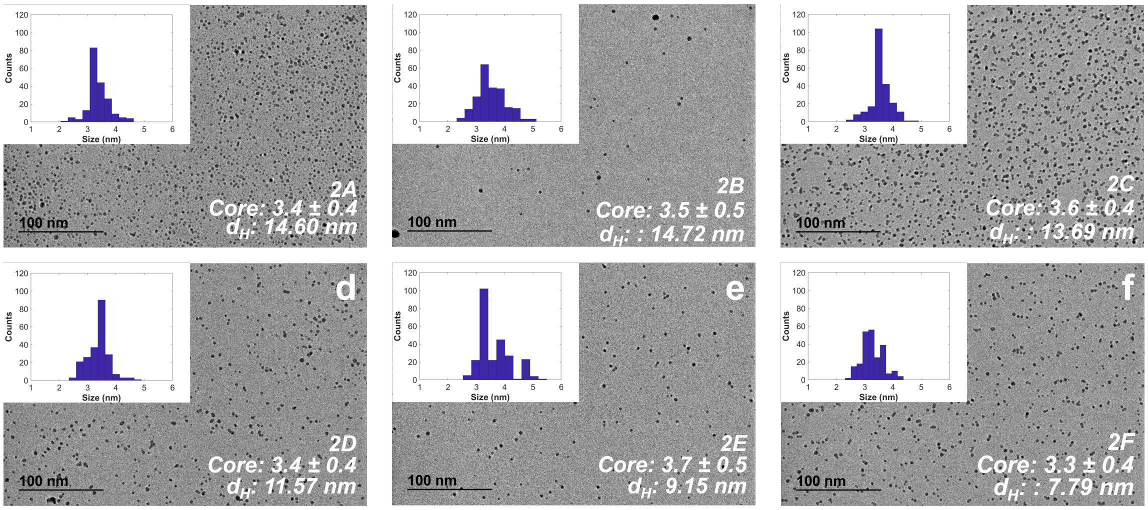

Figure S6: Representative transmission electron micrographs of 2kDa PEGSH NP samples 2A, 2B, 2C, 2D, 2E, and 2F. Histograms of core size distribution are included in the upper left hand corner of each image.

Gel Characterization

The moduli of the gels were characterized by small-amplitude oscillatory shear rheology, as described in the main text. Representative frequency sweeps for gels of each crosslink density/average mesh size are shown in Fig. S7(a-c). The low-frequency portion of the storage modulus was fit to a line (Fig. S7(d-f)), and the y-intercept of each fit was taken as the zero-frequency storage modulus $G'_{(0)}$ that was then used to calculate the gel's average mesh size.

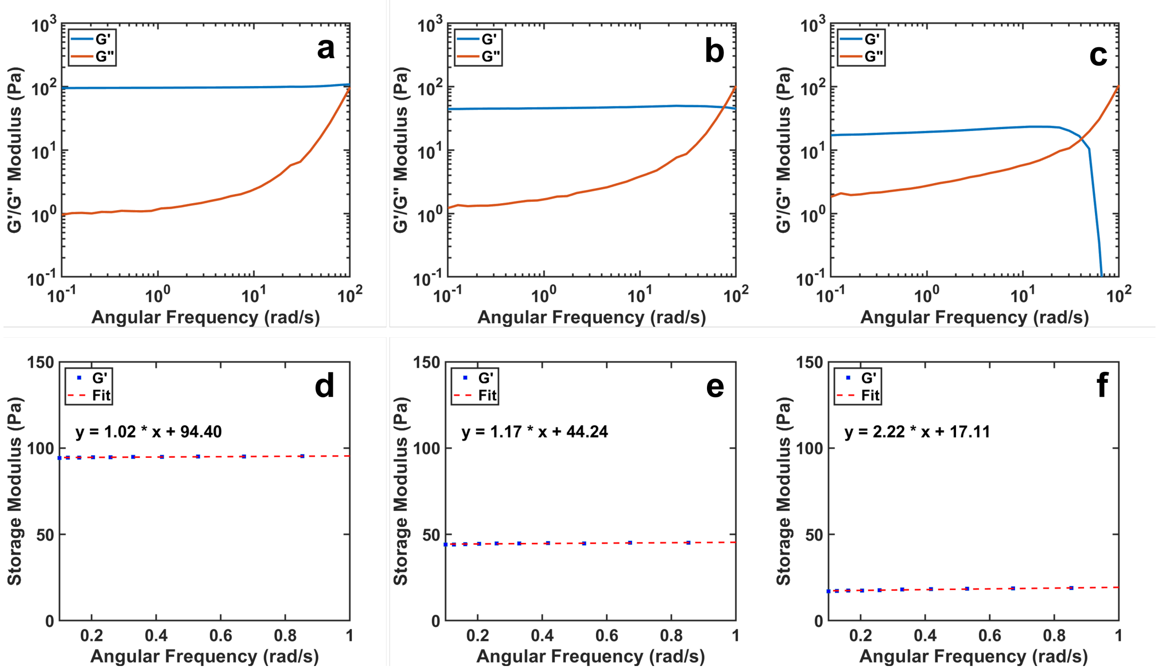

Figure S7: (a-c) Frequency sweeps conducted at 0.5% strain from 100 rad/s to 0.1 rad/sec, and (d-f) linear fits of the storage modulus in the low-frequency plateau for (a,d) the 35 nm gel, (b,e) the 45 nm gel, and (c,f) the 62 nm gel. The y-intercept from the linear fit was taken as $G'_{(0)}$ and used to calculate the gel's average mesh size as described in the main text.

Comparison of Diffusion in Gels vs. Free Solution

To highlight the role that the gel plays in the diffusion process, the data from Fig. 3 in the main text are re-plotted in Fig. S8, below, with an added line indicating the diffusion coefficients that would be measured for particles of each hydrodynamic diameter in free solution. As seen in this figure, the diffusion coefficients measured in the gels were always at least a factor of two smaller than those for the same particles in free solution, with larger deviation measured for particles with higher effective grafting densities.

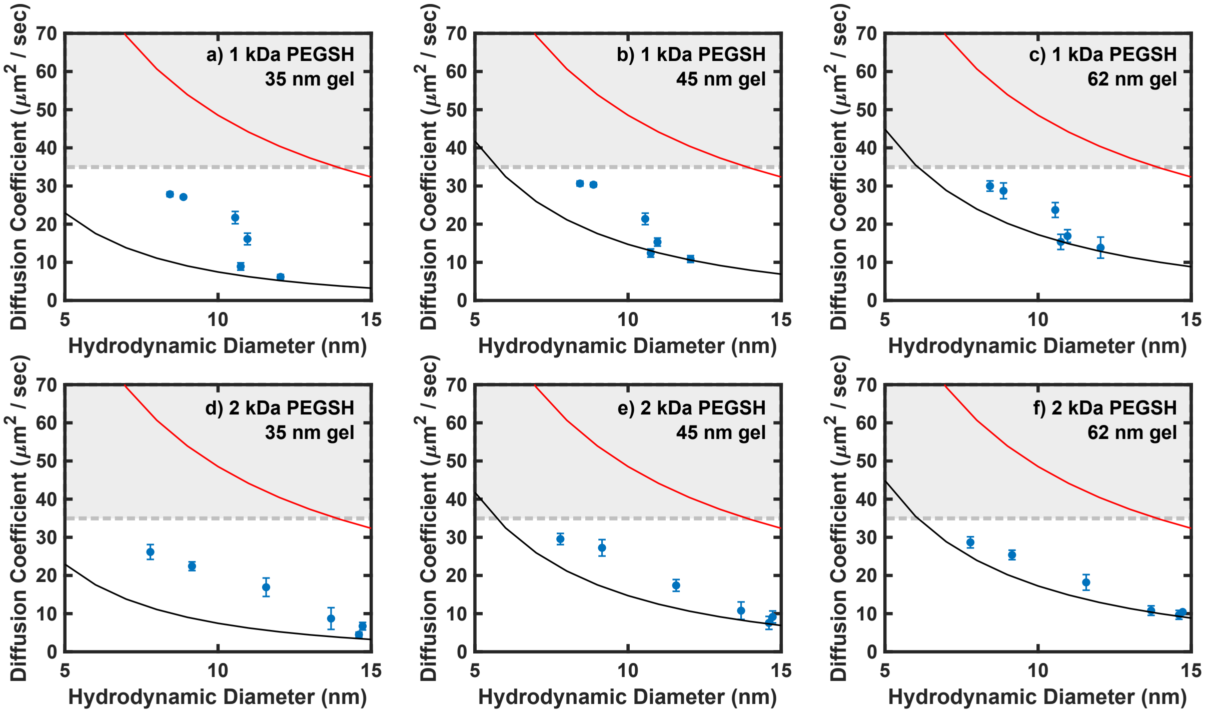

Figure S8: Diffusion coefficients of NPs with (a-c) 1 kDa ligands and (d-f) 2 kDa ligands in gels with average mesh sizes of (a,d) 35 nm, (b,e) 45 nm, and (c,f) 62 nm. The black line on each plot represents the diffusion coefficient predicted from fits to our prior data on densely-grafted particles,[S1] while the red line indicates the diffusion coefficients for particles with each hydrodynamic diameter in free solution. Areas shaded in grey indicate diffusion constants larger than the maximum diffusion coefficient plotted in Fig. 3.

Diffusion Coefficients of Densely-Grafted Particles

As noted in the main text, fits to our previous data on particles with densely-packed ligand shells were used to predict the effective size of the nanoparticles in the gels. In our prior work, the best fit of the relationship between nanoparticle diameter and diffusion coefficient was obtained using Cai and Rubinstein's hopping model,[S1,S2] and we thus use this model to provide a quantitative comparison of the behavior of particles with low effective grafting densities to those expected in the high grafting density limit. In this model, the diffusion coefficient D of a particle of diameter d is given by

$$D = \frac{\xi^2}{\tau_0 N_x^2 \phi} \left(\frac{b N_x^{1/2}}{d} \right) e^{\frac{-d^2}{N_x b^2}} \tag{3}$$

where ξ is the correlation length of the gel, τ_0 is the monomer relaxation time, N_x is the number of Kuhn monomers between crosslinks, b is the Kuhn monomer length (approx. 1.6 nm for polyacrylamide with $C_\infty = 8.5$[S3]), and ϕ is the polymer volume fraction. In our prior work,[S1] we fit data for NPs with fully-loaded ligand shells to determine values of both ξ^2/τ_0 and N_x in gels with average mesh sizes of 36 nm, 40 nm, 51 nm, and 60 nm. In that analysis, the values of N_x generally increased with average mesh size but exhibited some scatter, suggesting some overfitting of the data. To address this point, we re-fit the data using values of N_x determined from the average molecular weight between crosslinks expected for each synthesis condition, which were comparable to the N_x values extracted from the original fits, and allowed only ξ^2/τ_0 to vary. ξ^2/τ_0 was then plotted against the experimentally-determined average gel mesh size, and a linear fit of this data (Fig. S9) allowed estimation of the value of ξ^2/τ_0 for the gels prepared in the present work. The resulting parameters used to calculate the predicted diffusion coefficients shown in the main text are summarized in Table S4.

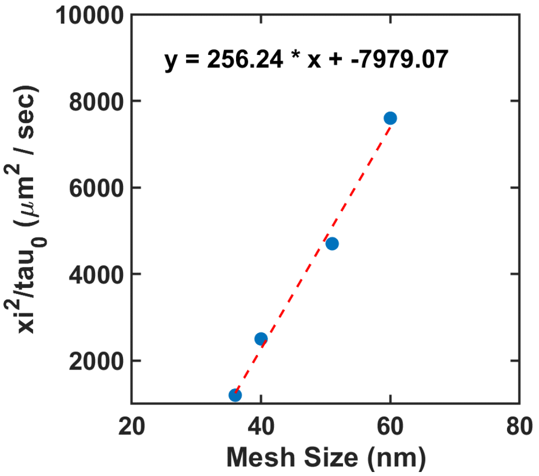

Figure S9: Linear fit of ξ^2/τ_0 values from our previous work[S1] used to estimate ξ^2/τ_0 values for the present gels.

Table S4: Interpolated hopping model parameters used to predict diffusion coefficients of NPs with fully-loaded ligand shells.

Gel Mesh Size (nm)	ξ^2/τ_0 (μm^2/sec)	N_x
35	1.0×10^3	54
45	3.6×10^3	81
62	7.9×10^3	145

References

(S1) Moncure, P.; Simon, Z.; Millstone, J.; Laaser, J. Relationship between Gel Mesh and Particle Size in Determining Nanoparticle Diffusion in Hydrogel Nanocomposites. *The Journal of Physical Chemistry B* **2022**, *126*, 4132 – 4142, DOI: 10.1021/acs.jpcb.2c00771.

(S2) Cai, L. H.; Panyukov, S.; Rubinstein, M. Hopping diffusion of nanoparticles in polymer matrices. *Macromolecules* **2015**, *48*, 847–862, DOI: 10.1021/ma501608x.

(S3) Bohdanecký, M.; Petrus, V.; Sedláček, B. Estimation of the characteristic ratio of polyacrylamide in water and in a mixed theta-solvent. *Die Makromolekulare Chemie* **1983**, *184*, 2061–2073, DOI: 10.1002/macp.1983.021841011.
